# Metformin use is associated with a reduced risk of acute appendicitis in Taiwanese patients with type 2 diabetes mellitus

**DOI:** 10.1038/s41598-021-91902-z

**Published:** 2021-06-11

**Authors:** Chin-Hsiao Tseng

**Affiliations:** 1 Department of Internal Medicine, National Taiwan University College of Medicine, Taipei, Taiwan; 2 Division of Endocrinology and Metabolism, Department of Internal Medicine, National Taiwan University Hospital, No. 7 Chung-Shan South Road, Taipei, 100 Taiwan; 3 Division of Environmental Health and Occupational Medicine of the National Health Research Institutes, Zhunan, Taiwan

**Keywords:** Endocrinology, Gastroenterology

## Abstract

This retrospective cohort study used the nationwide database of Taiwan’s National Health Insurance to investigate whether metformin would reduce the risk of acute appendicitis in patients with type 2 diabetes mellitus. We first identified 423,949 patients newly diagnosed of diabetes from 1999 to 2005. After excluding patients having type 1 diabetes mellitus, missing data, previous history of acute appendicitis, aged < 15 years, aged > 80 years and followed up for < 6 months, 338,172 ever users and 21,861 never users of metformin were followed up from January 1, 2006 until December 31, 2011. Incidence of acute appendicitis was estimated for never users, ever users and subgroups (divided by median, tertiles and quartiles, respectively) of dose–response indicators including cumulative duration (months), cumulative dose (mg) and average daily dose (mg/day) of metformin therapy. We used Cox regression incorporated with the inverse probability of treatment weighting using propensity score to estimate the overall hazard ratio for ever versus never users, and the hazard ratios for subgroups of dose–response indicators versus never users. Results showed that new-onset acute appendicitis was diagnosed in 1558 ever users and 179 never users during follow-up. The incidence was 98.15 per 100,000 person-years in ever users and was 189.48 per 100,000 person-years in never users. The overall hazard ratio (95% confidence interval) of 0.514 (0.441–0.600) suggested a lower risk of acute appendicitis associated with metformin use. A dose–response pattern was consistently observed in the analyses of different subgroups of dose–response indicators and the reduced risk associated with metformin use was consistently observed in various sensitivity analyses. An average daily dose of 1000–1500 mg/day can significantly reduce the risk by > 50%. The benefit did not differ between different formulations of metformin, and the estimated hazard ratio for conventional/immediate-release metformin versus never users was 0.516 (0.441–0.603) and was 0.509 (0.421–0.615) for prolonged/slow-release metformin versus never users. It is concluded that metformin use is associated with a reduced risk of acute appendicitis in patients with type 2 diabetes mellitus.

## Introduction

Acute appendicitis is a common acute inflammatory disease affecting the appendix, a hollow organ located at the tip of the cecum^1–3^. Obstruction of the appendiceal orifice caused by infection, lymphoid hyperplasia, fecaliths or tumor (either benign or malignant) is thought to be the likely pathophysiology leading to the acute inflammation of appendix^1^. Bacterial overgrowth, ischemia, necrosis and perforation can lead to acute peritonitis^1^. In the USA, acute appendicitis mostly occurs in youngsters aged 10–20 years with a sex ratio of male-to-female of 1.4 and the estimated lifetime risk of acute appendicitis in men was 8.6% and in women was 6.7%^2^. Appendectomy by surgical operation is a standard treatment, but treatment with antibiotics or endoscopic retrograde appendicitis therapy can be used in selected patients^3^.

Metformin is used as the first-line oral antidiabetic drug in the treatment of hyperglycemia in patients with type 2 diabetes mellitus because of its multiple pleiotropic benefits beyond glycemic control^4^. More than 150 million diabetes patients are taking the drug over the world^5^. Metformin can distribute to various tissues including the gastrointestinal tracts of stomach, small intestine, colon and appendix^6^. It has been demonstrated that metformin may exert anti-inflammatory, anti-microbial, anti-atherosclerotic, anti-neoplastic, anti-aging and immune modulating actions^7,8^. Our previous observational studies did suggest that diabetes patients who used metformin, in comparison to non-users, may have a lower risk of colorectal cancer^9,10^, pulmonary tuberculosis infection^11^, *Helicobacter pylori* infection^12^, inflammatory bowel disease^13^ and hemorrhoids^14^.

To the best of our knowledge, there has not been any previous study that investigates the effect of metformin on the risk of acute appendicitis, a common and potentially preventable disease. This study explored whether metformin could affect the risk of acute appendicitis in patients with type 2 diabetes mellitus.

## Materials and methods

### The National Health Insurance in Taiwan

Since March 1, 1995, Taiwan started to implement a compulsory healthcare system, the so-called National Health Insurance (NHI). More than 99% of the Taiwan’s population is covered by the NHI; and all hospitals and more than 93% of all medical settings in Taiwan provide medical care for the insurants of the NHI. Computer files that include records of disease diagnoses, drug prescriptions and clinical procedures should be submitted for the purpose of reimbursement. The database can be used for academic research if approved after ethics review. This study was approved with consent waiver by the Research Ethics Committee of the National Health Research Institutes (approval number 99274). In accordance to local regulations, all personal information needs to be de-identified before the release of the database for the protection of privacy. Therefore, informed consent is not necessary. All methods in the study were performed in accordance with the relevant guidelines and regulations.

### Definitions of diabetes and appendicitis

Throughout the study period, the database used the International Classification of Diseases, Ninth Revision, Clinical Modification (ICD-9-CM) as the disease coding system. Accordingly, 250.XX were the codes used for a diagnosis of diabetes mellitus. Acute appendicitis was coded 540 and the operation code for appendectomy was 47.0.

### Study population

**Figure 1:**
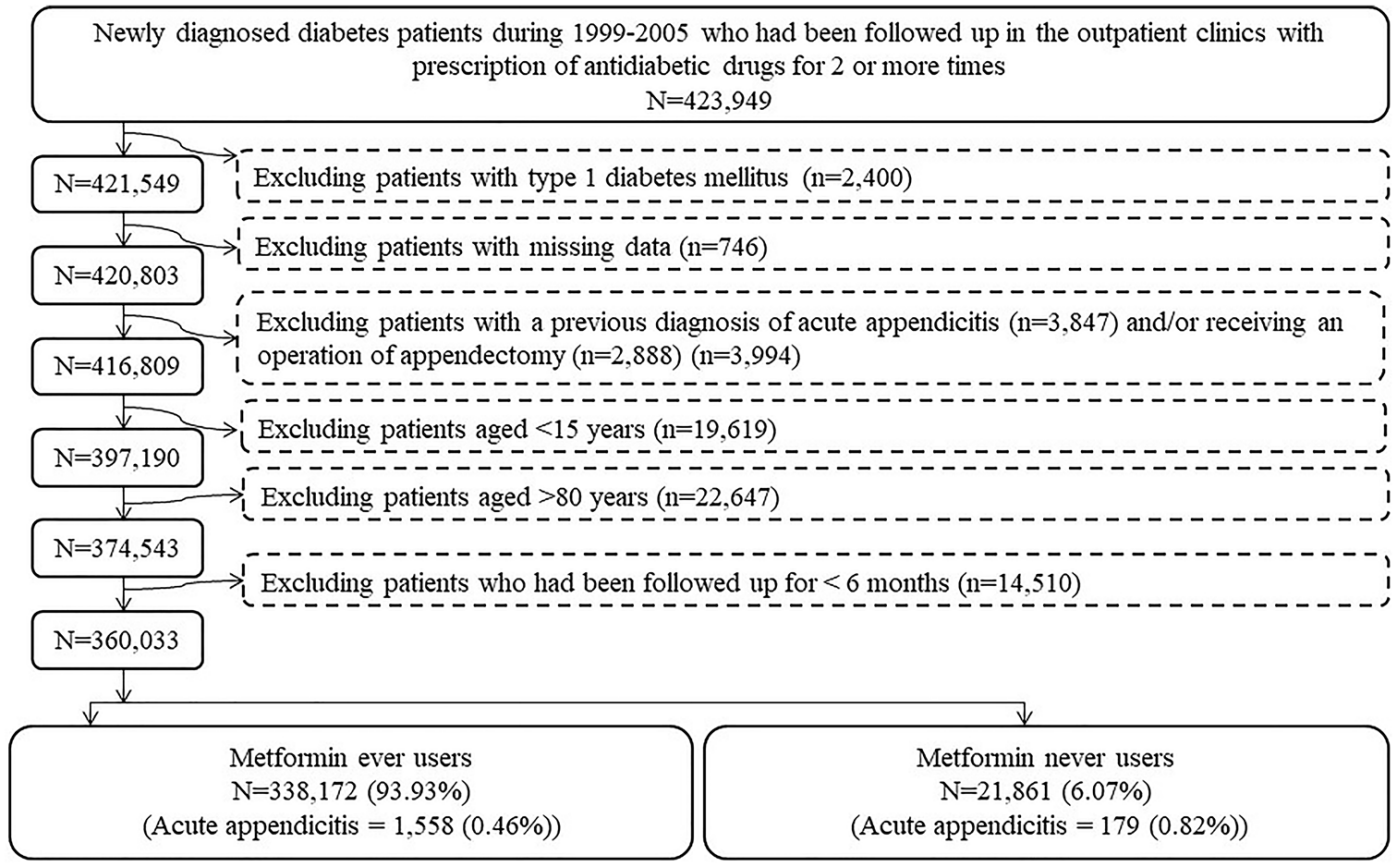
The procedures followed in enrolling ever users and never users of metformin from Taiwan’s National Health Insurance database.

Figure 1 is the flowchart that shows the procedures followed step-bey-step in the enrollment of metformin ever users and never users used for analyses in the study. At first, we identified from the database 423,949 patients who were newly diagnosed of diabetes from 1999 to 2005 and having been prescribed antidiabetic drugs in the outpatient clinics for at least two times. We then excluded the following ineligible patients: (1) type 1 diabetes (n = 2400), (2) missing data (n = 746), (3) patients who had a diagnosis of acute appendicitis and/or received an operation of appendectomy before the start of follow-up (n = 3994), and (4) patients aged < 15 years (n = 19,619), (5) patients aged > 80 years (n = 22,647), and (6) patients who had been followed up for < 6 months (n = 14,510). Data from patients with less than 6 months of follow-up were excluded because of the following considerations: (1) These patients followed up for a short period of time might have represented those who were not definite cases of diabetes mellitus or whose diabetes status could be handled well by non-pharmacological approaches after enrollment. (2) These patients might have a short life expectancy after diabetes diagnosis because of some other causes of mortality (e.g., cancer, accident, heart attack or stroke etc.) and the inclusion of them might have included inappropriate follow-up time in the calculation of person-years. (3) In consideration of biological plausibility in the assessment of a cause-effect relationship, there should always be a latent period for an outcome to happen after a certain exposure. Thus, the outcome, i.e., acute appendicitis, that occurred after a short period of follow-up might not be really related to the exposure under investigation, i.e., metformin or other antidiabetic drugs. In brief, the inclusion of patients followed up for a short period of time, say < 6 months, might have included inappropriate cases, violated biological plausibility and introduced inappropriate calculation of follow-up person-years. As a result, 260,033 patients (338,172 ever users of metformin and 21,861 never users of metformin) were included in the analyses of the study.

### Covariates

Some basic data, medications used by the patients and disease diagnoses were retrieved from the database and treated as potential confounders. Basic data included age, sex, occupation and living region. Occupation was divided into four classes according to the Bureau of NHI: (1) civil servants, teachers, employees of governmental or private businesses, professionals and technicians; (2) people without a specific employer, self-employed people and seamen; (3) farmers and fishermen; and (4) low-income families supported by social welfare and veterans. The living regions of the patients were classified into the following five categories according to the locations of the branch offices of the Bureau of NHI in different geographical regions: Taipei, Northern, Central, Southern, and Kao-Ping/Eastern.

Medications used by the patients were divided into two subgroups of antidiabetic drugs and medications commonly used by diabetes patients. Antidiabetic drugs included insulin, sulfonylurea, meglitinide, acarbose, rosiglitazone and pioglitazone. Medications commonly used by diabetes patients included angiotensin-converting enzyme inhibitors/angiotensin receptor blockers, calcium channel blockers, statins, fibrates and aspirin.

Disease diagnoses were divided into the following three categories: major comorbidities of diabetes, diabetes complications, and common comorbidities and potential risk factors. These disease diagnoses were selected because they might have a potential correlation with either the exposure or the outcome or because they might affect the patients’ life expectancy, leading to a shortened follow-up duration and a biased estimate of person-years in the calculation of incidence^13^. Disease diagnoses that might require the long-term use of antibiotics, steroid and anti-inflammatory drugs were especially considered because these drugs might have an impact on the risk of acute appendicitis. Major comorbidities of diabetes included hypertension, dyslipidemia and obesity. Diabetes complications included nephropathy, eye diseases, diabetic polyneuropathy, stroke, ischemic heart disease and peripheral arterial disease. Common comorbidities and potential risk factors included chronic obstructive pulmonary disease (a surrogate for smoking), tobacco abuse, alcohol-related diagnoses, cancer, heart failure, Parkinson’s disease, dementia, head injury, valvular heart disease, gingival and periodontal diseases, pneumonia, osteoporosis, arthropathies and related disorders, psoriasis and similar disorders, dorsopathies, liver cirrhosis, other chronic non-alcoholic liver diseases, hepatitis B virus infection, hepatitis C virus infection, human immunodeficiency virus infection, organ transplantation, *Helicobacter pylori* infection (041.86), diverticula of intestine (562) and peptic ulcer site unspecified (533). Except for the last three disease diagnoses, the ICD-9-CM codes for the others can be seen in a previous paper^13^.

Some previous studies have assessed the accuracy of the ICD-9-CM codes labelled in the NHI database for various disease diagnoses^15,16^. The codes of 250.XX used for the diagnosis of diabetes mellitus have a sensitivity of 90.9% and a positive predictive value of 90.2%^15^. In another study, moderate to substantial Kappa values ranging from 0.55 to 0.86 were noted for the agreements between claim data and medical records^16^.

### Statistical analyses

Statistical analyses were conducted by using version 9.4 of SAS statistical software (SAS Institute, Cary, NC). *P* < 0.05 was considered as having statistical significance.

Stuart argued against the use of hypothesis tests and *P*-values as a measure of balance assessment and recommended the use of standardized difference^17^. Unlike the *P* values of statistical tests of hypothesis that are sensitive to sample size, standardized difference is not so influenced and it allows quantitative comparison of the balance in measured baseline characteristics, either continuous or dichotomous, between the treated and untreated subjects in a sample^18^. Therefore, standardized difference between ever and never users of metformin calculated according to Austin and Stuart^18^ was used to evaluate the balance of characteristics. Although there is still no consensus on the value of a standardized difference to indicate meaningful confounding, some investigators used a threshold value exceeding 10% to indicate meaningful imbalance in baseline covariates between the treated and untreated groups^18^.

The median, tertiles and quartiles of cumulative duration (expressed in months), cumulative dose (expressed in mg) and average daily dose (expressed in mg/day) of metformin therapy were calculated and used as dose–response indicators. Incidence density of acute appendicitis was calculated with regards to metformin exposure in never users, ever users, and subgroups of dose–response indicators categorized by median, tertiles and quartiles, respectively. Follow-up started on January 1, 2006. The case number of patients who were newly diagnosed of acute appendicitis after the start of follow-up was the incidence numerator. The incidence denominator was the time of follow-up expressed in person-years. This was calculated from the start of follow-up until whichever of the following events occurred first, up to the date of December 31, 2011: a new diagnosis of acute appendicitis, death or the last available record in the reimbursement database.

Cumulative incidence functions of acute appendicitis with regards to metformin exposure were plotted for never users and ever users and their difference was tested by Gray’s test.

Propensity scores (PS) were estimated by logistic regression with independent variables that included the date of enrollment and the characteristics listed in Table 1. Some unmeasured factors (e.g., the changes of treatment guidelines, the improvement of therapeutic modalities, the prolongation of life expectancy and the introduction of newer classes of antidiabetic drugs) evolving during the long inclusion period might have been partially adjusted for by including the date of enrollment in the estimation of PS. Cox regression incorporated with the inverse probability of treatment weighting (IPTW) using the PS was used to estimate hazard ratios and their 95% confidence intervals. Austin recommended this regression method to reduce confounding resulting from the differences in characteristics between compared groups^19^. In the main analyses, the overall hazard ratio for ever users versus never users of metformin was estimated; and for the evaluation of a dose–response relationship, the hazard ratios for different subgroups of cumulative duration, cumulative dose and average daily dose of metformin therapy divided by median, tertiles and quartiles, respectively, were estimated by comparing to the never users.

**Table 1:** Characteristics in never users of metformin and ever users of metformin.

	Never users of metformin (n = 21,861)		Ever users of metformin (n = 338,172)		Standardized difference
	n	%	n	%	
**Basic data**					
Age* (years)	62.14 ± 11.62		58.36 ± 11.38		− 35.09
Sex (men)	12,182	55.72	179,180	52.98	− 5.90
Occupation					
I	8104	37.07	132,081	39.06	
II	3953	18.08	73,146	21.63	9.20
III	5031	23.01	70,696	20.91	− 4.96
IV	4773	21.83	62,249	18.41	− 9.39
Living region					
Taipei	7407	33.88	114,176	33.76	
Northern	2291	10.48	41,209	12.19	5.69
Central	3824	17.49	59,927	17.72	0.56
Southern	3806	17.41	54,766	16.19	− 3.27
Kao-Ping and Eastern	4533	20.74	68,094	20.14	− 1.08
**Major comorbidities of diabetes**					
Hypertension	16,733	76.54	239,424	70.80	− 14.18
Dyslipidemia	13,128	60.05	230,801	68.25	18.38
Obesity	461	2.11	14,820	4.38	13.02
**Diabetes complications**					
Nephropathy	6099	27.90	58,150	17.20	− 29.69
Eye diseases	2015	9.22	49,555	14.65	17.37
Diabetic polyneuropathy	2260	10.34	56,446	16.69	19.44
Stroke	6231	28.50	72,543	21.45	− 18.16
Ischemic heart disease	9339	42.72	124,029	36.68	− 13.90
Peripheral arterial disease	3710	16.97	57,303	16.94	− 0.76
**Antidiabetic drugs**					
Insulin	1929	8.82	7119	2.11	− 33.24
Sulfonylurea	15,570	71.22	218,049	64.48	− 8.95
Meglitinide	1930	8.83	12,267	3.63	− 23.78
Acarbose	2540	11.62	16,727	4.95	− 24.06
Rosiglitazone	635	2.90	14,517	4.29	8.21
Pioglitazone	526	2.41	7942	2.35	0.59
**Medications commonly used by diabetes patients**					
Angiotensin converting enzyme inhibitor/angiotensin receptor blocker	13,551	61.99	198,402	58.67	− 7.78
Calcium channel blocker	12,996	59.45	174,293	51.54	− 17.32
Statins	8563	39.17	149,362	44.17	10.69
Fibrates	5996	27.43	107,971	31.93	10.26
Aspirin	11,076	50.67	163,989	48.49	− 5.23
**Common comorbidities and potential risk factors**					
Chronic obstructive pulmonary disease	9767	44.68	139,189	41.16	− 8.43
Tobacco abuse	323	1.48	6653	1.97	4.01
Alcohol-related diagnoses	1328	6.07	17,851	5.28	− 4.31
Cancer	3151	14.41	33,306	9.85	− 16.04
Heart failure	4037	18.47	41,192	12.18	− 20.14
Parkinson’s disease	676	3.09	5731	1.69	− 10.58
Dementia	1390	6.36	14,512	4.29	− 10.99
Head injury	291	1.33	3920	1.16	− 2.00
Valvular heart disease	2306	10.55	24,208	7.16	− 13.83
Gingival and periodontal diseases	16,686	76.33	269,881	79.81	8.58
Pneumonia	2708	12.39	29,495	8.72	− 14.73
Osteoporosis	4579	20.95	59,743	17.67	− 9.47
Arthropathies and related disorders	15,299	69.98	231,862	68.56	− 3.63
Psoriasis and similar disorders	470	2.15	7621	2.25	0.64
Dorsopathies	15,047	68.83	236,822	70.03	2.41
Liver cirrhosis	1507	6.89	12,817	3.79	− 15.96
Other chronic non-alcoholic liver diseases	1792	8.20	30,673	9.07	3.11
Hepatitis B virus infection	512	2.34	5939	1.76	− 5.19
Hepatitis C virus infection	1145	5.24	12,293	3.64	− 8.97
Human immunodeficiency virus infection	16	0.07	179	0.05	− 0.92
Organ transplantation	164	0.75	570	0.17	− 11.32
*Helicobacter pylori* infection	138	0.63	1857	0.55	− 1.23
Diverticula of intestine	239	1.09	2894	0.86	− 3.04
Peptic ulcer site unspecified	8696	39.78	122,252	36.15	− 8.83

*Age is shown as “mean ± standard deviation”.

The classification of occupation can be seen in “Materials and Methods”.

In order to examine the consistency of the findings in more restricted subgroups, we estimated the following hazard ratios for ever users versus never users as sensitivity analyses: (1) patients were censored when four months have elapsed since the last prescription; (2) patients receiving other antidiabetic drugs before the initiation of metformin were excluded (thus the carry-over effect of other antidiabetic drugs could be excluded); (3) patients who had been followed up for less than twelve months were excluded; (4) patients who had a metformin treatment duration less than twelve months were excluded; (5) patients who were enrolled during 1999–2002 were analyzed; (6) patients who were enrolled during 2003–2005 were analyzed; (7) patients receiving two consecutive metformin prescriptions spanning more than four months were excluded (The Bureau of NHI allows a maximum duration of three months for each drug prescription. Therefore, these patients might have been irregularly followed up at the outpatient clinics and had a delayed drug refill for metformin for at least one month); (8) patients having been treated with incretin-based therapies during follow-up were excluded (This is to exclude the potential impact of incretin-based therapies which might happen to be prescribed during follow-up because the first incretin-based therapy was not reimbursed by the NHI in Taiwan until after 2009. Furthermore, the use of dipeptidyl peptidase 4 inhibitors has been shown to be associated with a change in the composition of gut microbiota^20^); (9) definition of acute appendicitis included an operation code of appendectomy in addition to a diagnostic code (the inclusion of an operation code might have reduced the potential risk of misdiagnosis of acute appendicitis); (10) patients aged < 50 years were included; (11) patients aged 50–64 years were included; and (12) patients aged ≥ 65 years were included.

According to the NHI database, only conventional/immediate-release formulation of metformin was available during the period of subject enrollment and the prolonged/slow-release formulation was first reimbursed by the NHI on August 1, 2006. To examine whether the effect of prolonged/slow-release formulation might not be similar to the conventional/immediate-release formulation, two additional sensitivity analyses were conducted by dividing ever users into two subgroups, i.e., those who had been prescribed prolonged/slow-release metformin after start of follow-up and those who had only been exposed to conventional/immediate-release formulation during the whole period of follow-up. The first model compared the two different subgroups to the referent group of never users of metformin (Model XIII). The second model was created after excluding never users and estimated the hazard ratio of prolonged/slow-release metformin to a referent group of conventional/immediate-release metformin (Model XIV). Because age may affect the use of metformin, an additional model was created without exclusion of patients because of their ages (Model XV) to examine whether the result would be consistent.

## Results

Table 1 shows the characteristics in never users and in ever users of metformin. Standardized difference > 10% was observed for age, hypertension, dyslipidemia, obesity, nephropathy, eye diseases, diabetic polyneuropathy, stroke, ischemic heart disease, insulin, meglitinide, acarbose, calcium channel blockers, statins, fibrates, cancer, heart failure, Parkinson’s disease, dementia, valvular heart disease, pneumonia, liver cirrhosis and organ transplantation. This justified the use of the method recommended by Austin^19^ in the estimation of hazard ratios.

**Figure 2:**
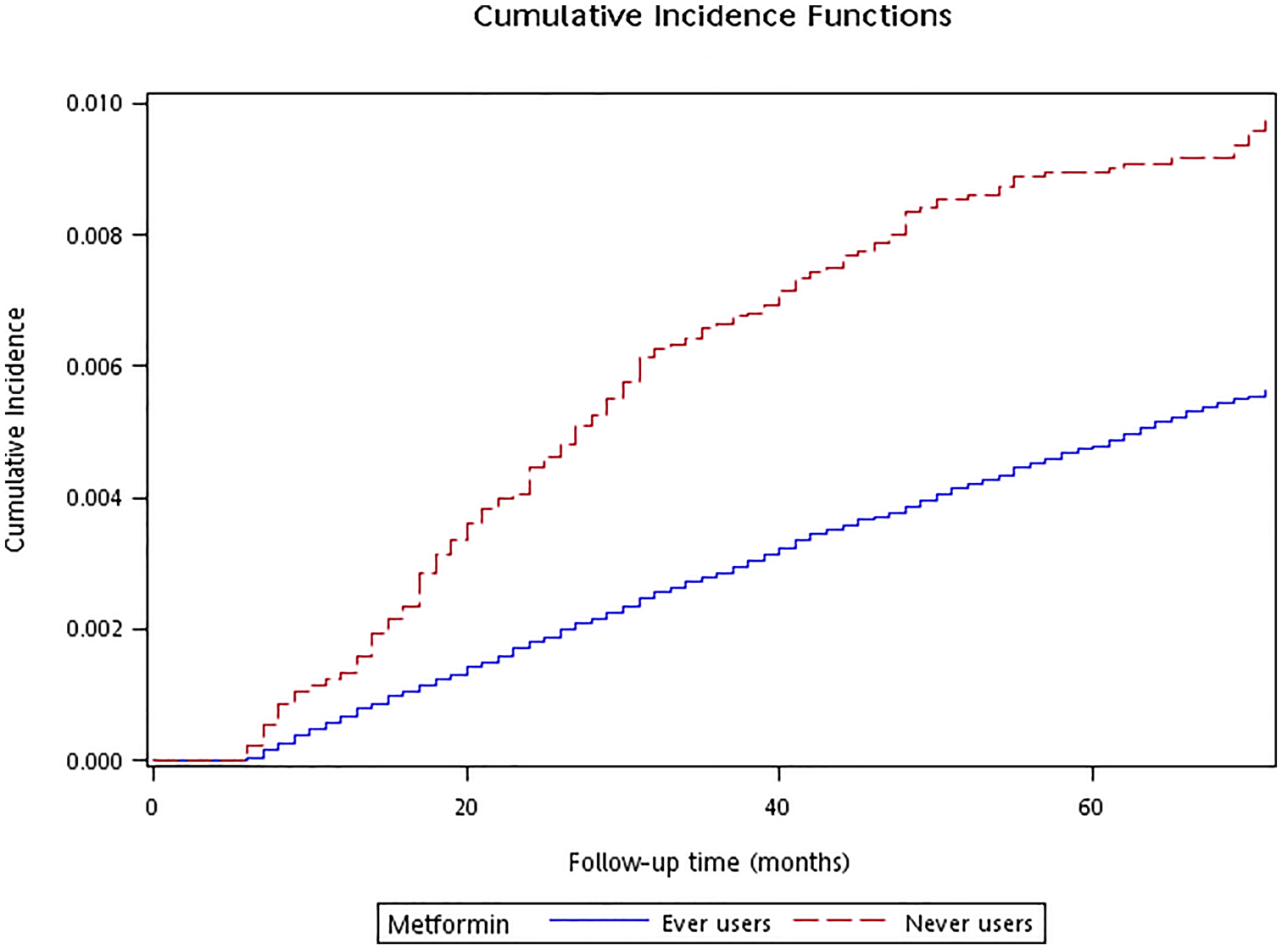
Cumulative incidence functions of acute appendicitis in never users and ever users of metformin (Gray’s test* P* < 0.01).

Figure 2 shows the cumulative incidence functions of acute appendicitis for never users and ever users. Never users of metformin obviously had a significantly higher cumulative incidence of acute appendicitis (Gray’s test *P* < 0.01).

Table 2 shows the incidence of acute appendicitis and the hazard ratios by metformin exposure. After a median follow-up duration of 5.3 years in never users and 5.5 years in ever users of metformin, the incidence rates of acute appendicitis were 189.48 and 98.15 per 100,000 person-years, respectively. The overall hazard ratio comparing ever versus never users suggested a significantly 49% lower risk associated with metformin use. Analyses of all dose–response indicators categorized by the cutoff values of median, tertiles and quartiles consistently showed an obvious dose–response pattern, indicating a cause-effect relationship in terms of either cumulative duration, cumulative dose or average daily dose of metformin therapy.

**Table 2:** Incidence rates of acute appendicitis in different subgroups of metformin exposure and hazard ratios comparing different subgroups of metformin users versus metformin never users.

Subgroups of metformin exposure	Cases of acute appendicitis identified during follow-up	Cases followed	Person-years	Incidence rate (per 100,000 person-years)	Hazard ratio	95% Confidence interval	*P* value
Never users	179	21,861	94,467.47	189.48	1.000		
Ever users	1558	338,172	1,587,344.40	98.15	0.514	(0.441–0.600)	< 0.0001
**Cumulative duration of metformin therapy (months)**							
Median cutoff							
Never users	179	21,861	94,467.47	189.48	1.000		
< 42.53	1066	169,047	652,768.90	163.30	0.850	(0.726–0.997)	0.0452
≥ 42.53	492	169,125	934,575.50	52.64	0.263	(0.221–0.312)	< 0.0001
Tertile cutoffs							
Never users	179	21,861	94,467.47	189.48	1.000		
< 26.93	751	111,549	386,965.78	194.07	1.000	(0.849–1.178)	0.9999
26.93–59.23	532	111,601	544,855.47	97.64	0.503	(0.425–0.596)	< 0.0001
≥ 59.23	275	115,022	655,523.15	41.95	0.208	(0.172–0.251)	< 0.0001
Quartile cutoffs							
Never users	179	21,861	94,467.47	189.48	1.000		
< 20.00	616	84,507	276,044.94	223.15	1.138	(0.962–1.345)	0.1326
20.00–42.53	450	84,540	376,723.96	119.45	0.619	(0.520–0.736)	< 0.0001
42.53–70.00	318	84,315	445,657.22	71.36	0.366	(0.304–0.439)	< 0.0001
≥ 70.00	174	84,810	488,918.28	35.59	0.178	(0.145–0.220)	< 0.0001
**Cumulative dose of metformin therapy (mg)**							
Median cutoff							
Never users	179	21,861	94,467.47	189.48	1.000		
< 1,336,500	1038	169,080	659,998.06	157.27	0.821	(0.700–0.962)	0.0147
≥ 1,336,500	520	169,092	927,346.34	56.07	0.283	(0.239–0.336)	< 0.0001
Tertile cutoffs							
Never users	179	21,861	94,467.47	189.48	1.000		
< 782,800	734	111,596	391,428.63	187.52	0.962	(0.816–1.134)	0.6453
782,800–2,006,400	524	111,597	548,188.10	95.59	0.494	(0.417–0.585)	< 0.0001
≥ 2,006,400	300	114,979	647,727.67	46.32	0.232	(0.193–0.279)	< 0.0001
Quartile cutoffs							
Never users	179	21,861	94,467.47	189.48	1.000		
< 555,500	595	84,524	279,080.75	213.20	1.088	(0.919–1.288)	0.3263
555,500–1,336,500	443	84,556	380,917.31	116.30	0.603	(0.507–0.718)	< 0.0001
1,336,500–2,494,000	329	84,537	445,507.23	73.85	0.381	(0.317–0.457)	< 0.0001
≥ 2,494,000	191	84,555	481,839.11	39.64	0.200	(0.163–0.246)	< 0.0001
**Average daily dose of metformin therapy (mg/day)**							
Median cutoff							
Never users	179	21,861	94,467.47	189.48	1.000		
< 1069.14	818	169,086	743,703.63	109.99	0.577	(0.491–0.678)	< 0.0001
≥ 1069.14	740	169,086	843,640.77	87.72	0.458	(0.389–0.540)	< 0.0001
Tertile cutoffs							
Never users	179	21,861	94,467.47	189.48	1.000		
< 965.97	541	111,597	486,232.24	111.26	0.584	(0.493–0.691)	< 0.0001
965.97–1275.23	541	111,599	524,562.94	103.13	0.541	(0.457–0.641)	< 0.0001
≥ 1275.23	476	114,976	576,549.21	82.56	0.433	(0.365–0.514)	< 0.0001
Quartile cutoffs							
Never users	179	21,861	94,467.47	189.48	1.000		
< 872.79	421	84,543	359,795.46	117.01	0.613	(0.514–0.730)	< 0.0001
872.79–1069.14	397	84,543	383,908.16	103.41	0.543	(0.455–0.647)	< 0.0001
1069.14–1403.17	389	84,543	418,859.39	92.87	0.487	(0.408–0.582)	< 0.0001
≥ 1403.17	351	84,543	424,781.39	82.63	0.433	(0.362–0.519)	< 0.0001

A significantly lower risk of acute appendicitis in ever users of metformin could be consistently shown in all sensitivity analyses (Table 3). Although the benefit might attenuate with increasing age, the lower risk associated with metformin use could be seen in all age groups (Models X, XI and XII) and would not be affected by including patients of all ages into analysis (Model XV). While comparing different formulations of metformin, it is evident that both the prolonged/slow-release formulation and the conventional/immediate-release formulation were significantly associated with a lower risk of acute appendicitis to the same extent (Models XIII and XIV).

**Table 3:** Sensitivity analyses.

Models/metformin use	Cases of acute appendicitis identified during follow-up	Cases followed	Person-years	Incidence rate (per 100,000 person-years)	Hazard ratio	95% Confidence interval	*P* value
**I. Patients were censored when four months have elapsed since the last prescription**							
Never users	179	21,861	94,467.47	189.48	1.000		
Ever users	1302	338,172	1,371,917.12	94.90	0.502	(0.430–0.587)	< 0.0001
**II. Patients receiving other antidiabetic drugs before the initiation of metformin were excluded**							
Never users	179	21,861	94,467.47	189.48	1.000		
Ever users	731	157,164	747,611.26	97.78	0.513	(0.436–0.604)	< 0.0001
**III. Patients who had been followed up for less than twelve months were excluded**							
Never users	151	20,489	93,443.98	161.59	1.000		
Ever users	1350	328,312	1,579,951.19	85.45	0.522	(0.441–0.618)	< 0.0001
**IV. Patients who had a metformin treatment duration less than twelve months were excluded**							
Never users	179	21,861	94,467.47	189.48	1.000		
Ever users	1172	286,039	1,427,290.77	82.11	0.424	(0.363–0.497)	< 0.0001
**V. Patients who were enrolled during 1999**–**2002 were analyzed**							
Never users	69	9672	40,842.35	168.94	1.000		
Ever users	832	186,526	890,661.14	93.41	0.548	(0.429–0.701)	< 0.0001
**VI. Patients who were enrolled during 2003**–**2005 were analyzed**							
Never users	110	12,189	53,625.12	205.13	1.000		
Ever users	726	151,646	696,683.26	104.21	0.506	(0.414–0.618)	< 0.0001
**VII. Patients receiving two consecutive metformin prescriptions spanning more than four months were excluded**							
Never users	179	21,861	94,467.47	189.48	1.000		
Ever users	405	102,675	455,350.92	88.94	0.468	(0.393–0.558)	< 0.0001
**VIII. Patients having been treated with incretin-based therapies during follow-up were excluded**							
Never users	175	20,543	88,412.14	197.94	1.000		
Ever users	1454	264,845	1,212,399.53	119.93	0.602	(0.515–0.704)	< 0.0001
**IX. Definition of acute appendicitis included an operation code of appendectomy in addition to a diagnostic code**							
Never users	124	21,861	94,602.32	131.08	1.000		
Ever users	1060	338,172	1,588,583.53	66.73	0.505	(0.419–0.608)	< 0.0001
**X. Patients aged < 50 years were included**							
Never users	39	3458	15,668.74	248.90	1.000		
Ever users	406	77,749	380,516.39	106.70	0.426	(0.307–0.592)	< 0.0001
**XI. Patients aged 50**–**64 years were included**							
Never users	70	8413	37,354.75	187.39	1.000		
Ever users	665	156,143	742,628.59	89.55	0.475	(0.371–0.607)	< 0.0001
**XII. Patients aged ≥ 65 years were included**							
Never users	70	9990	41,443.99	168.90	1.000		
Ever users	487	104,280	464,199.42	104.91	0.617	(0.480–0.793)	0.0002
**XIII. Metformin formulation**							
Never users	179	21,861	94,467.47	189.48	1.000		
Metformin: Conventional/immediate-release	1294	280,557	1,314,729.16	98.42	0.516	(0.441–0.603)	< 0.0001
Metformin: Prolonged/slow-release	264	57,615	272,615.23	96.84	0.509	(0.421–0.615)	< 0.0001
**XIV. After excluding never users of metformin**							
Ever users: Conventional/immediate-release	1294	280,557	1,314,729.16	98.42	1.000		
Ever users: Prolonged/slow-release	264	57,615	272,615.23	96.84	0.982	(0.861–1.121)	0.7914
**XV. No exclusion of patients because of their ages**							
Never users	255	27,306	106,315.25	239.85	1.000		
Ever users	1857	366,224	1,651,312.11	112.46	0.473	(0.415–0.539)	< 0.0001

## Discussion

This is the first study that showed a lower risk of acute appendicitis associated with the use of metformin in patients with type 2 diabetes mellitus. The results were consistent in the main analyses (Table 2) and the sensitivity analyses (Table 3); and a dose–response relationship could be demonstrated for all dose–response indicators by using different cutoffs (Table 2). The results did not differ significantly between different formulations of metformin (Table 3, Models XIII and XIV).

Although not yet researched, the availability of metformin in the appendix^6^ and the anti-inflammatory, anti-microbial and immune modulatory properties of metformin^7,8^ could partly explain such a reduced risk. Gut immune-microbe interaction may play an important role in the development of acute appendicitis^21^ and a prospective clinical trial investigating the relationship between gut microbiota and acute appendicitis is being conducted^22^. *Fusobacteria* infection has been linked to acute appendicitis^23^ and *Akkermansia muciniphila* (a mucin-degrading bacterium that colonizes in the mucus layer and improves intestinal barrier function)^24^ is inversely associated with the severity of acute appendicitis^23^. It is interesting that metformin may reduce the proliferation of *Fusobacteria*^25^ and induce the proliferation of *Akkermansia muciniphila* in the gut^20^.

Another possible explanation is related to the effect of metformin on intestinal motility. Metformin is well-known for its adverse effects on the gastrointestinal tract including diarrhea, nausea, vomiting, abdominal pain, flatulence, bloating and retching, which can occur in 20–30% of the patients treated with metformin^26,27^. Most of these adverse events are minor and may attenuate after prolonged use and metformin would have to be discontinued because of severe gastrointestinal symptoms in only approximately 5% of the users^27^. Because appendicoliths may potentially lead to acute appendicitis^1,28^, the promotion of intestinal motility resulting from these adverse effects of metformin may reduce the risk of acute appendicitis because of the reduced occurrence of appendix obstruction by appendicoliths. Because prolonged/slow-release metformin may only have minimal improvement of gastrointestinal intolerance than conventional/immediate-release formulations^29^, it is not surprising that the use of either formulation was associated with a similarly lower risk of acute appendicitis (Table 3, Models XIII and XIV) taking the increased gut motility as a potential mechanism.

There are some clinical implications in the present study. First, although confirmation of the findings is necessary, the lower risk of acute appendicitis associated with metformin use observed in the present study can be viewed as an extra bonus among other pleiotropic effects of metformin in addition to its glucose lowering effect. This may significantly reduce the clinical and economical burdens of acute appendicitis. Second, it would be wise to continue the use of metformin in patients without any contraindication when addition of other antidiabetic drugs is required for better glycemic control because the potential mechanisms might not be dependent on glycemic control. Furthermore, the dose–response effect observed in association with cumulative duration and cumulative dose of metformin therapy (Table 2) and the significant effect seen only after at least a continuous use for more than one year (Model IV, Table 3) favored the continuation of the use of metformin. Third, because a higher average daily dose might have a better effect of risk reduction of acute appendicitis (Table 2), metformin should be titrated to the highest tolerable daily dose of up to at least approximately 1000–1500 mg/day to obtain a maximal effect of > 50% risk reduction (Table 2, average daily dose of metformin therapy).

There are some limitations in the present study. First, measurement data of potential confounders, biochemical profiles and genetic factors were not available in the database and only diagnostic codes could be used for adjustment. Second, although we tried to balance the baseline characteristics between ever users and never users of metformin by the IPTW method using propensity scores in order to avoid confounding by indication, it was not sure whether residual confounding might exist because unmeasured confounders could never be adjusted for by statistical methods. However, if unmeasured confounders are associated with measured confounders, adjustment for measured confounders may also adjust for the unmeasured ones^17,30^. Third, we could not exclude the possibility of other interpretations. For example, it is not known whether other non-metformin medications would increase the risk of acute appendicitis in never users of metformin rather than a true effect of risk reduction of metformin. Furthermore, the reason for the decision to prescribe non-metformin medications, over metformin, among never users might also be the reason for the increased risk of acute appendicitis. Fourth, misclassification of disease diagnoses was possible in the database. However, because nondifferential misclassification would only lead to a bias toward the null, the estimated hazard ratios were expected to be underestimated. The consistency of the findings in the main analyses (Table 2) and the sensitivity analyses (Table 3) suggested that the lower risk of acute appendicitis associated with metformin use might be robust. Fifth, knowledge of absolute risk reduction and number needed to treat is important for decision making and clinical application^31^. As the incidence of acute appendicitis was low, the absolute risk reduction calculated was too small (179/21861–1558/338172 = 0.36%) and the number needed to treat (the reciprocal of absolute risk reduction) of 279 was too large. Therefore, whether the use of metformin to prevent acute appendicitis is cost-effective remains to be investigated, especially in non-diabetes people. Some earlier clinical trials provided evidence of beneficial effects of metformin for weight reduction and enhanced insulin sensitivity in normoglycemic morbidly obese adolescents^32^ and in non-diabetic morbidly obese adults^33^. However, whether the benefits of metformin use in non-diabetes and non-obese people can outweigh its potential adverse events is not known, especially in the elderly people.

There are several strengths associated with the use of this large population-based database and the design of the study. First, the high coverage rate of the NHI and the enrollment of a large sample size of all diabetes patients during a long period of time (1999–2005) and the long follow-up duration (from 2006 to 2011) would surely avoid the problems of selection bias and the lack of statistical power. Therefore, the findings could be readily applied to the whole population. Second, self-reporting bias and recall bias could be prevented by using existing medical records. Third, prevalent user bias and immortal time bias were carefully addressed during the stages of enrollment of study cohort and procedures of statistical analyses. Prevalent user bias could be prevented by enrolling only patients with a new diagnosis of diabetes and new users of antidiabetic drugs. Immortal time bias may result from inappropriate assignment of treatment status and/or miscalculation of follow-up time. In the present study we included only patients with a definite diagnosis of diabetes mellitus because the patients had to been prescribed antidiabetic drugs for at least 2 times (Fig. 1). Because longitudinal information of drug prescription was complete and available in the NHI database, misclassification of treatment status with metformin was unlikely and the calculation of the dose–response indicators could be less biased. We did not include the following immortal times in the calculation of follow-up person-years: (1) the duration from the time of diabetes diagnosis to the time when antidiabetic drugs were started; and (2) the initial short follow-up period of < 6 months. Furthermore, the immortal time between the date of hospital discharge and the date when discharged drugs are refilled is not a problem in Taiwan because all discharge drugs can be readily obtained on the date of hospital discharge. Fourth, because cost-sharing is low and much expense can be waived in some patients (e.g., veterans, patients with low income, and patients receiving refills of drugs for chronic diseases) in the NHI healthcare system, detection bias as a result of varying socioeconomic status is less of a problem in Taiwan.

In summary, this is the first observational study suggesting a lower risk of acute appendicitis associated with metformin use, disregarding the different formulations of metformin. Such a benefit seems to be dose-responsive and may be maximized to > 50% risk reduction by increasing the average daily dose to 1000–1500 mg/day or prolonging the cumulative duration or cumulative dose of metformin therapy. Because of the inherent limitations associated with the observational study design, it is necessary to have further confirmatory studies and clinical trials are welcome to provide a definite conclusion. The cost-effectiveness of the use of metformin in the prevention of acute appendicitis is not known, especially in elderly non-diabetes and non-obese people. The findings of the present study at least support that it is appropriate to recommend the use of metformin as the first-line antidiabetic drug given its various beneficial effects beyond glycemic control including a lower risk of acute appendicitis.
